# Lived Experiences of Patients with Chronic Kidney Disease Receiving Hemodialysis in Felege Hiwot Comprehensive Specialized Hospital, Northwest Ethiopia

**DOI:** 10.1155/2021/6637272

**Published:** 2021-08-25

**Authors:** Hailemariam Tadesse, Hordofa Gutema, Yosef Wasihun, Samuel Dagne, Yonatan Menber, Pammela Petrucka, Netsanet Fentahun

**Affiliations:** ^1^Felege-Hiwot Comprehensive Specialized Hospital, Bahir Dar, Ethiopia; ^2^School of Public Health, College of Medicine and Health Sciences, Bahir Dar University, Bahir Dar, Ethiopia; ^3^College of Nursing, University of Saskatchewan, Saskatoon, Canada

## Abstract

**Purpose:**

Chronic kidney disease is a challenging disease and global public health problem. The burden of chronic kidney disease and hemodialysis is increasing in Ethiopia, but few studies explored the lived experiences of chronic kidney disease patients receiving hemodialysis. This study explored the lived experiences of chronic kidney disease patients receiving hemodialysis, in the Felege Hiwot Comprehensive Specialized Hospital, Bahir Dar City, Northwest Ethiopia, 2019.

**Methods:**

A phenomenological study design was conducted with 12 chronic kidney disease patients receiving hemodialysis between September 1 and October 30, 2019. A purposive sampling technique was used to select participants, and a semistructured in-depth interview guide was used to collect the data. The investigators audio-taped the interviews and then transcribed them verbatim. Finally, the transcribed data were imported to Atlas.ti™-7 software for coding, and then, thematic analysis was done. Transferability, dependability, credibility, and conformability were embedded to ensure data quality.

**Results:**

In this study, six major themes were emerged: (1) the seriousness of the disease, (2) challenges to get hemodialysis, (3) financial constraint, (4) restricted life, (5) feeling of dependency, and (6) psychological impacts.

**Conclusion:**

The restrictive nature of the disease affects a participant's financial status which makes it challenging to obtain the service and increases feelings of dependency. These circumstances impact the psychology of the participants. We would recommend that every patient with hemodialysis needs social and psychological support. We would also recommend the need to extend the study to other areas of the country to confirm or disconfirm the findings.

## 1. Introduction

Chronic kidney disease (CKD) is defined as abnormal kidney composition or function persisting for longer than 3 months [1]. CKD is a global public health problem due to a rise in common comorbidities such as diabetes, hypertension, and obesity [2]. By definition, CKD is a progressive, permanent impairment in the renal task, which results in metabolic, fluid, and electrolyte imbalances in the body [3].

CKD is classified into five stages based on the level of glomerular filtration rate [3]. Stage 5 is considered as nonreversible kidney failure, so dialysis or a kidney transplant will likely be needed to prolong life [4]. Any stage of CKD is linked with increased risks of cardiovascular disease, premature mortality, and/or decreased quality of life [5].

Globally, more than 500 million people are affected by CKD [1, 6]. The incidence of CKD has continued to rise at a rate of 8% per year and consumes up to 2% of the overall global health expenditure [7]. More than one million people in the world with severe CKD are being treated with some form of therapy, with 90% of these patients living in developed countries [5]. There is a considerable rise in the number of CKD patients who are taking hemodialysis management [8, 9]. Hemodialysis is expensive and inaccessible to many in developing countries, and the increased nonadherence rate of supportive treatment is also a common problem in CKD patients due to financial constraints [1, 10–12].

Annually, in Ethiopia, deaths related to renal disease exceeded 12,000 people (1.47% of total deaths) [13]. In Ethiopia, hemodialysis is inaccessible and unaffordable for the majority of the target population. Each CKD patient's expenses for each hemodialysis treatment are more than 1000 Ethiopian Birr (30 US$) [14]. Currently, in Ethiopia, there are few dialysis centers only found in public hospitals [15].

CKD patients live with significant dietary restrictions, which results in an impairment in health [16, 17].

Studying lived experience of CKD patients on hemodialysis will help to improve services by connecting health professionals, researchers, and policy-makers to design solutions for encountered problems. Since limited qualitative researches were done previously, this study will be used to fill the gaps by exploring the real lived experience of patients on hemodialysis.

Therefore, this study explored the lived experiences of CKD patients receiving hemodialysis, in Felege Hiwot Comprehensive Specialized Hospital, Bahir Dar City, Northwest Ethiopia.

## 2. Materials and Methods

### 2.1. Study Setting and Design

The phenomenological study design was conducted between September 1 and October 30, 2019, in Felege Hiwot Comprehensive Specialized Hospital (FHCSH), Bahir Dar City, Amhara Region, Northwest Ethiopia. FHCSH is found 560 km Northwest of Addis Ababa, the Capital City of Ethiopia. It provides health services for more than 5 million people. According to FHCSH 2018/2019 report, the hospital provided a hemodialysis service per year for over 2800 CKD patients. These patients got 2-3 times hemodialysis service in a week. Hemodialysis service is given only in FHCSH in Bahir Dar City.

### 2.2. Study Participants

The study participants were patients with CKD and use hemodialysis treatment for more than three months in the FHCSH hemodialysis center. Chronic kidney disease with hemodialysis patients aged 18 years and above were included in this study. Twelve CKD patients with hemodialysis were purposively selected for an in-depth interview (IDI) that included both males and females. A purposive sampling technique was used to select the study participants. The criteria for recruiting reflect the potential relevance of the participants in delivering a wealth of information about the lived experience of CKD with hemodialysis. In addition, the maximum variation technique was applied to include participants with variations of characteristics like educational status, residence, age, marital status, distance from the dialysis center, and capability of paying for the service.

The sample size was determined based on theoretical saturation, and 12 participants were involved. Saturation was declared by listing some important findings. Participants were identified from the hemodialysis (HD) center patient registration book and their schedule of HD procedure with the aid of the HD center coordinator.

### 2.3. Data Collection Methods and Tools

The study adopted a semistructured in-depth interview guide from the evidence/literature [18–20]. It was pretested from three samples in Gondar Comprehensive Specialized Hospital which is found 169 km away from the study area. The guide was developed first in English, translated into Amharic (local language) to collect the data, and then translated back to English to check the consistency.

In-depth interviews were employed to collect the data in the hospital in a separate room. The principal investigator conducted twelve in-depth interviews (IDIs). The principal investigator (PI) conducted face-to-face interviews using the IDI guide. Newly emerging insights and questions were added for clarification and depth in the following IDI. The data were recorded using a tape recorder, and the investigator took notes including memos of the participants' behavior and contextual aspects to augment the data with the record. The IDIs took a minimum of 45 minutes.

During data collection, participants were engaged in a dialogue-like approach, first asking the participant a question, then listening attentively until the individual completes his/her idea, and then probing based on the response of the participant.

### 2.4. Data Analysis

All IDIs were transcribed verbatim after a minimum of three-repeat listening. The transcribed documents were imported into Atlas.ti™ version 7 for coding and analysis. The investigators then coded the respondent's words, phrases, sentences, and memos relevant to the area of the study. They systematically coded raw data openly and categorized the subthemes under their respective themes. Then, they created nonrepetitive central themes that were constructed based on the formed categories. Finally, investigators also cross-checked the themes that emerged after analysis with the raw data and respective quotes in each category of the themes. Direct quotes of the participants were included in the write-up of the findings.

### 2.5. Data Quality Assurance

Developed data collection tools were pretested in a similar context to maximize the validity. Probing and multiple data sources were employed to collect the data. At the onset of the study, bracketing the preconceptions of the investigator was employed to minimize the investigator's bias and the risk of reactivity by the participants. Also, the literature review was delayed until the data collection and analysis to minimize bias. The emerging findings were shared with experienced qualitative researchers for peer debriefing before synthesizing the final outputs. Transferability was achieved by describing the study setting, sample, and data collection procedure clearly and in detail. Dependability was obtained to increase the consistency of the study and could be repeated. Confirmability of the study was ensured by the recording of every activity of the participants during the time of the interview.

## 3. Results

Twelve participants were involved in this study. Ten of the participants were male, and the mean age of the participants was 37 years with a range of 24 to 50 years. Six of the participants stopped working due to the disease, and five participants were using hemodialysis for more than two years (Table 1).

### 3.1. Lived Experiences of CKD Patients with Hemodialysis

The study participants in FHCSH of Bahir Dar City reported their lived experience of CKD with hemodialysis as the seriousness of the disease, challenges to get hemodialysis, financial constraints, restricted life, feeling of dependency, and psychological influence.

**Theme 1 .**

The seriousness of the disease.

CKD is recognized as a devastating and life-threatening condition in the theme seriousness of the disease. The conditions that make it too difficult to live with the disease were the poor outcome, lifelong treatment, restriction in full participation in their social life and daily activity, and expenses associated with the treatment.

*...it's hard to describe this disease. It's a terrible disease. It hurts my economy, family, neighbors … a generally devastating disease. I don't expect to be cured. If I don't get dialysis, the pain will be worsening.* (Participant 12*).*

Lifelong treatment of the disease and limited intake of foods and beverages were described as the most horrible thing by CKD patients when comparing CKD with other diseases they knew.

*Living with this disease is more difficult than living with other diseases. Different individuals said that AIDS was hard, but AIDS is easy when compared to chronic kidney disease. They eat what they want, they drink what they need, they live freely, but I can't eat what I get, I can't drink what I get, even water doesn't allow too much.* (Participant 2)

**Theme 2 .**

Challenges of getting hemodialysis.

Inaccessibility of hemodialysis was the main issue that was mentioned as a deterrent. Most participants travel long distances to access hemodialysis service, two or three times a week. Such situations make it difficult for them to reach a hemodialysis center according to their dialysis schedule. Travel costs and cost of treatment for hemodialysis and other supportive care were described as challenging.

*…I separated from my family because of the disease. The hemodialysis center didn't found in the area where I live, but, now I live here with a rent home. Life is always a struggle but living with this disease is challenging. I don't know how I live the remaining life in such a way. Generally, I can't express the effect of this disease on my life, his face changed and his tear was drawn.* (Participant 5)

The commonly reported issue was the frequent breakdown of the dialysis machines, fluctuation of electrical power, and absence of filtered water, and some supply which plays a great role in poor adherence to the treatment. It exposes them to wait long queues and then stay for more than a week without getting the service.

*I faced a problem light interruption when I was on the bed. My blood in the line of the dialysis machine was discarded due to clotting and I waited for an additional 2-3 hours on a bed.* (Participant 11)

Long procedure time is one of the problems mentioned as a challenge by most participants during hemodialysis service. Most of the participants spend their time on HD by sleeping, using Facebook, and watching TV.

*It is a long time that seems greater than one full day. It is challenging. If you compare workplace time and hemodialysis procedure time, in the workplace, you entered 8:30 and went out for lunch at 12:30, the time run unconsciously, but here spending time on dialysis bed is boring and looks like a double day.* (Participant 3)

**Theme 3 .**

Financial constraint.

Financial constraints are challenging issue that prevents participants from accessing hemodialysis service. Almost all participants believed that the service they obtain from the dialysis center was uncertain due to financial constraints. Most participants decreased the frequency of hemodialysis service that they got within a week and also they usually ignore additional ordered supportive medications due to financial constraints.

*I did hemodialysis once per week due to the affordability issue of the service. My family was unable to cover 2-3 times hemodialysis payment, and I always think about cost and depressed me.* (Participant 7)

Most participants sell their available assets (i.e., goat, ox, house, car, etc.) to afford their treatment; others borrow or beg from families, relatives, and/or others.

*I have no remaining asset in my hand. Even I sold my car. All my properties were lost due to the disease. Still, now I survived with my own, but for the future, I fear dependency for others which is the most I hate in my life.* (Participant 8)

Participants feel the disease reaches far beyond themselves.

*… as I mentioned earlier [angrily], the disease made me paralyze in my economy, then it affects my family, relatives, and neighbors.* (Participant 12)

**Theme 4 .**

Restricted life.

Almost all of the study participants describe their grief in how CKD has placed limitations on their lives. Even though participants feel as they live a limited life, they strictly adhere to fluid and diet restrictions.

*The disease made me a poor man and when the restriction of food and drink is added, it makes the problem double burdened. As you know when I have no resources I can't select food and drinks. Because I had only limited alternatives, I always eat and drink what I had in my hand without selection. This makes the disease worsen, that is why I said earlier double burden.* (Participant 8)

The experience of fatigue was described as reducing an individual's potential and energy which results in a decreased capacity to perform daily activities. Such restricted activities affect social life and quality of life. Most participants described their feelings of loss due to fatigue.

*... the disease does not make you mobile, work as you want and get income, because the disease is hard. It tends to hurt your bones, hmm..., it's exhausting, your energy is decreased like a baby walk... you look as someone drunk.* (Participant 9)

While the participants have different roles in their families and the community, many of them find their role and performance have been disturbed since living with CKD. After the occurrence of the disease, the majority of the participants' plans were distorted.

*As a human being, I had the plan to get my Ph.D. In addition to this, I had plans to build a house, writing books, and do other activities, but everything remains a wish. Everything goes to a cliff. Now I have no hope to conduct those plans, everything is dead, just it downgrades your potential and makes your family hopeless. The only thing that I worried about is how to survive.* (Participant 4)

The majority of the participants described the consequences of the disease on their marriage in terms of their marital and community relationships.

*I expect that she was not happy in her life [sexual life]. When I see this, I say this disease has an impact on marriage. I know she lost the love from me because I am suffering from a disease and I can't give love as she wants at this time (lough), the disease is so challenging.* (participant 4)

Some participants described experiencing discrimination concerning participation in community activities. Additionally, the relationships are often impacted due to the potential for kidney donation from their family or relatives affecting their relationships.

*Now I stopped all social activities totally what I did before. Stigma and discrimination are high Among CKD patients than HIV/ADIS patients that I observed. Even my family members discriminate against me, so no social life at all, nobody cares about you and helps you… (cried). Rather than giving me solution and strength, most of the people around me make me hurt by different bad speeches.* (Participant 4)

Most participants believed that hemodialysis is a blessing from God. They tried to use their faith as a coping mechanism while they live in a stressful situation.

*I believe in God. …I believe in God as I receive the grace of God to see what tomorrow brings to me.* (Participant 9)

Some participants believed the disease is given by God to punish them as the consequence of sin; hence, participants may be discriminated against in their participation in religious activities. Participants were prevented from fasting, praying together, and baptisms.

*In Muslims, I have to bow down three times a day but I can't honor. Because of this, I do not apply all the services of faith. If I don't do that I'll be very bored. Of course, I cannot do everything, I must leave it to the Creator.* (participant 7).

**Theme 5 .**

Feeling of dependency.

Almost all the participants described as they rely on someone with financial, physical, or social while they live with this disease. Each activity of the participant needs family or social support.

Participant 8 stated the following.

*I can't sit in the toilet, without the help of others. Since it was a matter of blood clots, my thighs' were swollen, sore all around, and I had reached the point where I couldn't do all the hygiene myself and use the toilet. So I suffered until I could no longer use the toilet in own and it was very hard. They told me that the dialysis was done with the support of the family.*

Most participants believed that they lived dependent on the machine.

*Normally it is good, which removed that dangerous pain... and give me hope of living. But when I think my life suspended on the machine is very painful. (*Participant 4)

*Sometimes I said thank you, God, because God will never give me a disease, without giving the medicines [saydegs aytalam], when I was in the dialysis center.* (Participant 12)

**Theme 6 .**

Psychological impacts.

Almost all participants have psychological problems related to body image and mental wellness. Body swelling, abdominal distention, and darkening of the face catalyzed the negative self-perception. Sleep disturbances, anxiety, and depression lead them to suicidal ideations or attempts.

*I was deciding to kill myself, to be free from hemodialysis. During that time, I was unable to move. There is no change after this disease happened in my life and I wish I could die. I feel lonely. It is boring to live with such a condition... [She cried].* (Participant 2)

Some of the participants live with anxiety over the poison of the serious disease; others experience worry about the loss of their potential.

Participant 9 described the following.

*I couldn't look at the things that you had and my moral is exhausted, I act like a 70 or 80-year old, I passed my time on the bed and I get embarrassed.*

## 4. Discussion

The current study explored the lived experience of CKD patients receiving hemodialysis in the FHCSH of the Amhara Region. According to the current study, the seriousness of the disease was reflected in the “horrible nature” of CKD as needing lifelong treatment, incurring extensive treatment costs and restriction in social life, lacking accessibility to hemodialysis services, and being restricted in intake of diet and fluids. This study finding was in line with the studies conducted in Nigeria [18] and India [21], where participants described the loss of their freedom to participate in different activities, restriction in diet, fatigue associated with a lack of strength and energy, and decreased ability to perform their jobs. In the current study, participants clearly described their immense sense of loss on multiple dimensions. This result consistent with studies conducted in Nigeria [18], Iran [22], Francisco [23], Georgia [4], and the USA [19], with those living with CKD, described their lives as being filled with pain and suffering, loss of self, and loss of independence.

According to this study, participants frequently experience a shortage of filtered water and repeated breakdowns of the dialysis machines due to frequent electrical power interruptions which are similarly reported in studies from Nigeria [18] and the USA [19].

The current study showed that the inaccessibility of the hemodialysis service in health is a barrier to participants making it difficult to reach the dialysis center on time. This finding was consistent with a study conducted in Nigeria [18] whereas a study conducted in New Zealand [24] contradicted this result where there is a provision of in-home service and satellite dialysis centers. Also, long procedure times (3-4 hours) were mentioned as a challenge in this study and supported by studies conducted in South Korea [25], New Zealand [24], and Australia [26].

This study showed that participants often opted for a reduction of hemodialysis sessions per week and missed prescribed drugs due to financial constraints related to transportation and lack of ability to work. This finding is similar to the study conducted in Nigeria [18], where financial constraints were mentioned as a major barrier to service adherence. In addition, participants spoke of the consumption of assets to pay for their treatments, which is also reported in studies conducted in Nigeria [18] and the USA [19].

This study revealed that the disease disturbed participants in the performance of their roles in families and community while impacting their ability to plan. Similar results were reported in studies conducted in India [21] and Iran [27].

The current study showed that social life complexities like marital relationship disturbance contribute to separation, segregation, and stigma by communities and even families. This finding is supported by a study conducted in Spain [28]. On the other hand, the current study showed that most participants have a strong religious base which is articulated in the belief that the treatment they obtain in the HD center was blessed by God, resonating with a study from Jordan [29]. Some participants, however, contradict the above finding stating that the disease was given by God to punish them (a consequence of sin) which limited their participation in religious activity. A study conducted in Nigeria [18] also reported that CKD patients had a negative sense toward faith and blamed their illness on spiritual factors or on “divine intervention”.

This study revealed participants' experience of multilevel dependencies on financial, physical, social support, and the hemodialysis machine while they live with this disease. This finding is supported by studies conducted in Nigeria [18], Iran [22], New York [30], and India [21]. The sense of machine dependency is also mentioned by studies conducted in Australia [26] and India [31].

Another important finding of the current study is the psychological influence of the disease. Self-image, stigma, and suicidal ideations are all considered within the rubric of mental distress by the participants. This complex and far-reaching result was consistent with the studies conducted in India [31] Nigeria [18], Iran [22], Greece [32], and the USA [19], where participants described feelings of isolation in the society, physical limitations, and fears of poor clinical results contributing to feelings of sadness, depression, and despair. Further, the lifelong (chronic) dimension of CKD along with the uncertainties with regard to the ability to continue treatments and afford the medications impacted significantly on psychological wellbeing. This finding is supported by studies conducted in Iran [27] and Sweden [33].

## 5. Conclusions

The lived experiences of CKD patients receiving hemodialysis were found to be broad, significantly negative narratives from the participants in this study. Whether discussing, the seriousness of the disease challenges to get hemodialysis, financial constraints, restricted life, feelings of dependency, or psychological influence was evident where there were many shared experiences. Inaccessibility of hemodialysis service was a major challenge and an extra expense. Restrictions from available foods and drinks were a double burden that limited participants from using an available resource. Financial incapability was another challenge raised due to the loss of income-generating activities. As a result, a feeling of dependency on others was reported as leading to the development of mental distress (psychological influence).

Based on this study, we would recommend the need to extend the study to other areas of the country to confirm or disconfirm the findings. Further, we would recommend that a longitudinal study be considered which would look at the psychosocial domains of CKD to build holistic programs (financial, social, psychological) and other interventions (i.e., universal health coverage; transportation subsidies) which are more responsive to the needs of this challenged population (Tables 2 and 3).

## Figures and Tables

**Table 1:** Sociodemographic characteristics of participants in FHCSH Hemodialysis Center, Bahir Dar City, Northwest Ethiopia, 2020.

Participants	Age range	Gender	Marital status	Educational status	Residence	Occupation	Duration of using hemodialysis in months
Participant 1	40–49	M	Married	Primary school	Rural	Farmer	8
Participant 2	30–39	F	Divorced	Cannot read and write	Rural	Merchant	8
Participant 3	20–29	M	Single	College and above	Urban	Gov't employee	9
Participant 4	40–49	M	Married	College and above	Urban	Gov't employee	20
Participant 5	40–49	M	Married	College and above	Urban	Gov't employee	24
Participant 6	30–39	M	Married	Secondary school	Urban	Merchant	36
Participant 7	20–29	M	Single	Secondary school	Rural	Family dependent	24
Participant 8	≥50	M	Married	Secondary school	Urban	Retired	72
Participant 9	40–49	M	Divorced	Secondary school	Urban	Merchant	36
Participant 10	20–29	M	Single	Secondary school	Urban	Family dependent	36
Participant 11	20–29	F	Single	College and above	Urban	Gov't employee	42
Participant 12	≥50	M	Married	College and above	Urban	Retired	36

**Table 2:** Sociodemographic characteristics of the participants.

S/n	Character	Response of the participant
1	Age	
2	Sex	
3	Marital status	
4	Educational status	
5	Occupation	
6	Source of income	
7	Monthly income in birr	
8	Year of CKD diagnoses	
9	Year of hemodialysis initiation	
10	Frequency of hemodialysis in a week	
11	Cause of the disease	

**Table 3:** Area to be observed.

S/n	Observational component
1	What looks like the reception
2	In what way the participant waits for the procedure on the reception
3	How many minutes the participant wait to get the service
4	Communication of participant with another patient
5	Communication of participant with the health care provider
6	The activity of participant immediately after completing the procedure
7	How looks the service

## Data Availability

The datasets supporting the conclusions of this article are included within the article.

## Abbreviations

CKD: Chronic kidney disease
ESRD: End-stage renal disease
FHCSH: Felege Hiwot Comprehensive Specialized Hospital
GFR: Glomerular filtration rate
HD: Hemodialysis.

## Additional Points

*Limitation.* The study shows the Inability to generalize this finding to related populations.

## Supplementary Materials

Supplementary Materials Supplement document 1: Filled Consolidated Criteria for Reporting Qualitative Studies (COREQ) checklist Supplement document 2: Information sheet and Consent form Supplement document 3: Interview Guide.
