# Supplementary material for: Lived Experiences of Patients with Chronic Kidney Disease Receiving Hemodialysis in Felege Hiwot Comprehensive Specialized Hospital, Northwest Ethiopia
Source: Int J Nephrol. 2021 Aug 25;2021:6637272. doi: 10.1155/2021/6637272 (PMC8410445; doi:10.1155/2021/6637272)
Supplement: Supplementary Materials — Supplement document 1: Filled Consolidated Criteria for Reporting Qualitative Studies (COREQ) checklist Supplement document 2: Information sheet and Consent form Supplement document 3: Interview Guide. [file 6637272.f1.zip › Supplement 1-Filled COREQ Checklist.docx]

| 1. Interviewer/facilitator: Hailemariam Tadesse 2. Credentials: MPH in Health Promotion 3. Occupation: Health Promotion Officer at Hospital 4. Gender: Male 5. Experience and training: He had previous qualitative research experience, trained qualitative research and certified. 6. Relationship established: Yes 7. Participant knowledge: Participants clearly understood about the personal goals and reasons for doing the research. 8. Interviewer characteristics: The interviewer was interested in the research topic 9. Methodological orientation and participant selection: phenomenology | |
| --- | --- |
| 10. Sampling | Purposive sampling technique used |
| 11. Method of approach | Participants approach was face-to-face. |
| 12. Sample size | Twelve IDI |
| 13. Non-participation | No refusal |
| 14. Setting of data collection | In the hospital from separated room |
| 15. Presence of non-participants | No |
| 16. Description of sample | The important characteristics of the sample was demographic data were included and the lived experience was explored in detail. |
| 17. Interview guide | A semi-structured in-depth interview guide was used which was pilot tested before the actual data collection. |
| 18. Repeat interviews | Yes, repetition was done as needed |
| 19. Audio/visual recording | Yes, audio was recorded |
| 20. Field notes | Yes, field notes made during the interview |
| 21. Duration | A minimum of 45 minute for each interview. |
| 22. Data saturation | Yes |
| 23. Transcripts returned | Yes |

| 24. Number of data coders | Three |
| --- | --- |
| 25. Description of the coding tree | Yes |
| 26. Derivation of themes | The themes were derived from the data. |
| 27. Software | ATLAS.ti 7 |
| 28. Participant checking | Yes |
| 29. Quotations presented | Yes |
| 30. Data and findings consistent | Yes |
| 31. Clarity of major themes | Yes |
| 32. Clarity of minor themes | Yes |

Downloaded from https://academic.oup.com/intqhc/article/19/6/349/1791966 by guest on 10 February 2021
