# Supplementary material for: Lived Experiences of Patients with Chronic Kidney Disease Receiving Hemodialysis in Felege Hiwot Comprehensive Specialized Hospital, Northwest Ethiopia
Source: Int J Nephrol. 2021 Aug 25;2021:6637272. doi: 10.1155/2021/6637272 (PMC8410445; doi:10.1155/2021/6637272)
Supplement: Supplementary Materials — Supplement document 1: Filled Consolidated Criteria for Reporting Qualitative Studies (COREQ) checklist Supplement document 2: Information sheet and Consent form Supplement document 3: Interview Guide. [file 6637272.f1.zip › Supplement 2-Information sheet and consent form.docx]

## **Information sheet and consent form**

**A. Participants’ information sheet**

Study Title: “Lived Experience of Patient with Chronic Kidney Disease on Hemodialysis in Felege-Hiwot Comprehensive Specialized Hospital, North-West Ethiopia”

I would like to invite you to participate in a research study on the lived experience of Patients with Chronic Kidney Disease on Hemodialysis of people who are accessing hemodialysis treatment. However, before you decide on whether to be involved you need to understand why this research is being done and what may be expected of you throughout the time of the study. Please take time to read the following piece of information carefully and ask any questions if anything is not clear to you or if you require further information. Feel free to decide whether you wish to take part or not.

The purpose of the study is to gain an insight into the experiences of chronic kidney disease patients with hemodialysis, in Felege Hiwot comprehensive specialized hospital, Bahir Dar, Northwest Ethiopia.

**Why have I been invited?**

You have been selected to participate in this study because you have chronic kidney disease and are receiving hemodialysis treatment in the Renal Unit of the Felege Hiwot comprehensive specialized hospital. Your name was randomly selected from all the possible patients that could be asked to be involved in the study.

**Do I have to take part?**

Participation in the research study is entirely voluntary. You are completely free to decide. This information sheet will describe the process of the research study and you are free to ask any further questions to enable you to make an informed decision on whether to participate or not. You will then be asked to sign a consent form if you agree to participate in the study. You are free to withdraw at any time, without giving a reason and this will not affect the standard of care or treatment you receive in the facility.

**What will happen to me if I take part?**

If you agree to participate in the research study, you will agree to take part in 2 interview sessions over one week. There will be two sessions of an interview with each interview lasting between 30 to 60 minutes a week. The interview will take place at a time and place which is convenient for you and it will be tape-recorded and securely saved in a data file which will be placed on a password-protected computer to which only the researcher will have access. Secondary data will be collected from your hospital records in the Renal Unit. The data will include your last date biochemistry tests such as the hemoglobin levels, serum urea, and albumin and creatinine levels in the blood.

**Will I receive any payment or expenses?**

For the time spent during each interview session, a travel cost of 100 birrs will be paid by the researcher as a reimbursement for your time and travel costs to attend the interview.

**What are the possible disadvantages and risks of taking part?**

This study should pose no known risks, discomfort, or inconvenience to you. However, should you find any aspect of the study inconvenient, please feel free to let me know and this will not in any way affect the quality of your care or treatment?

**What are the possible benefits of taking part?**

Participating in the research may not be of any benefit to you but the findings from this study will help in generating evidence that will be used in improving the quality of care, which is also vital to improve the intervention provided to those CKD patients. Providing better access and sustainability of dialysis treatment to chronic kidney disease patients and enabling the healthcare practitioners in improving the care they render to the patients.

**Will my taking part in the study be kept confidential?**

All the information you provide during the interviews will be used for this study only. Your responses, details contact will be stored safely and confidentially on a password-protected computer, which will be accessed only by the researcher. Your responses to the questions will be transcribed and analyzed to understand and describe your experiences. After the completion of the study, all your information will be stored secretly. In addition, your responses will be coded secretly using pseudonyms instead of using your real names to ensure the confidentiality of your identity. No one will know that you have taken part in this study unless you choose to tell them.

**What will happen if I don’t carry on with the study?**

If you withdraw from the study we will destroy all your identifiable samples/ tape-recorded interviews, but we will need to use the data collected up to your withdrawal.

**What will happen to the results of the research study?**

The study findings will be published in academic journals. Any quotes from your interview that are used in any report/publication will not identify you unless you give specific consent. If you would like to be provided with a summary of the study findings and recommendations and these will be sent to you. Or if you decide later you want to see the results you can contact me directly at any time to ask for a copy of the findings.

Any further questions about the research or you want more information please contact:

Hayilemariam Tadese MPH student in Bahir Dar University

Emai:hayilemariamt@gmail.com

Mobile: 0918313169

**Thank you for taking the time to read this information**

**B. Consent Form**

Participant Identification Number _________

Hello Dear participants

I am ____________________, who is part of this research project entitled “Lived Experience of Patient with Chronic Kidney Disease on Hemodialysis in Felege-Hiwot Comprehensive Specialized Hospital, North-West Ethiopia”. I would like to ask some questions on the lived experience of a CKD patient with hemodialysis in FHCSH, Bahir Dar. We assure you that whatever information you provide will only be used for this research and will not be made available to anyone. During the interview, I use an audio recorder as means of data collection but your name and involvement in the study will remain confidential. The information you provide is used as part of the final study or journal publication but nothing is exposed to your privacy. We also assure you that the interview process will not bring any harm to you and your family. Your participation is voluntary. If you choose not to answer a particular question, that is your right. You are also permitted to withdraw any time from the study when you feel uncomfortable with it. I appreciate you too much for your willingness and support to respond to the interview. Therefore we together can do something positive towards CKD patients with hemodialysis outcomes. Finally, it is my happiness to forward you deepest gratitude in advance for the kind cooperation you are going to have during the interview by giving your time with genuine information to me. Once again, I am assuring you, by any means, you're confidentiality will not be broken and be kept secret and the data generated will be used for this research only.

The purpose of the study and confidentiality procedures has been explained to me and I put my Signature with my consent:

A). Agree ---- 2). Disagree----

- Date of Interview ------------------------------Time Started------------Time Finished-----------
- Name of participant --------------------------- Signature ---------------
- Interviewer’s Name ---------------------------Signature ----------------

**Thank you very much!**
