# Supplementary material for: Lived Experiences of Patients with Chronic Kidney Disease Receiving Hemodialysis in Felege Hiwot Comprehensive Specialized Hospital, Northwest Ethiopia
Source: Int J Nephrol. 2021 Aug 25;2021:6637272. doi: 10.1155/2021/6637272 (PMC8410445; doi:10.1155/2021/6637272)
Supplement: Supplementary Materials — Supplement document 1: Filled Consolidated Criteria for Reporting Qualitative Studies (COREQ) checklist Supplement document 2: Information sheet and Consent form Supplement document 3: Interview Guide. [file 6637272.f1.zip › Supplement 3-Interview Guide.docx]

## **Interview Guide**

**A. Socio-demographic character of the participants**

| **s/n** | **Character** | **Response of the participant** |
| --- | --- | --- |
| 1 | Age |  |
| 2 | Sex |  |
| 3 | Marital status |  |
| 4 | Educational status |  |
| 5 | Occupation |  |
| 6 | Source of income |  |
| 7 | Monthly income in birr |  |
| 8 | Year of CKD diagnoses |  |
| 9 | Year of hemodialysis initiation |  |
| 10 | Frequency of hemodialysis in a week |  |
| 11 | Cause of the disease |  |

**B. Interview guide**

1. What are your role in the family and social situation?
2. How do you express CKD?
3. How do you express the feeling while you live with CKD?
4. What change CKD had brought to your life?

Probe: (daily activity, religious activity, social life, working aria)

1. What things make it difficult to live with CKD?

Probe: (To accessing treatment, attend based on schedule)

1. What symptoms do you experience?

Probe: How do you express its feeling?

1. How do you express hemodialysis machine?
2. How do you express the feeling while you live with hemodialysis treatment?

Probe: What do you feel before, during and after the procedure?

1. What things influence you to attend the dialysis schedule?

Probe: (cost, workplace, your culture, the interest of your family.)

1. What do you feel when you miss one schedule of dialysis?

Probe :( What is the reason to miss the schedule?) 1

1. How do you express the care that you receive in the unit?

**C. Area to be observed**

| **S/N** | **Observational component** |
| --- | --- |
| 1 | What looks like the reception |
| 2 | In what way the participant waits for the procedure on the reception |
| 3 | How many minutes the participant wait to get the service |
| 4 | Communication of participant with another patient |
| 5 | Communication of participant with the health care provider |
| 6 | The activity of participant immediately after completing the procedure |
| 7 | How looks the service |
